# Pregnancy termination for fetal abnormality: are health professionals’ perceptions of women’s coping congruent with women’s accounts?

**DOI:** 10.1186/s12884-017-1238-3

**Published:** 2017-02-08

**Authors:** Caroline Lafarge, Kathryn Mitchell, Andrew C. G. Breeze, Pauline Fox

**Affiliations:** 1 School of Human and Social Sciences, University of West London, Paragon, Boston Manor Road, Brentford, TW8 9GA UK; 2 The Office of the Vice-Chancellor, University of Derby, Kedleston Road, Derby, DE22 1GB UK; 3 Fetal Medicine Unit, Leeds Teaching Hospitals NHS Trust, Leeds, LS1 3EX UK

**Keywords:** UK, Termination of pregnancy, Fetal abnormality, Coping and coping strategies, Health professionals, Congruence

## Abstract

**Background:**

Pregnancy termination for fetal abnormality (TFA) may have profound psychological consequences for those involved. Evidence suggests that women’s experience of care influences their psychological adjustment to TFA and that they greatly value compassionate healthcare. Caring for women in these circumstances presents challenges for health professionals, which may relate to their understanding of women’s experience. This qualitative study examined health professionals’ perceptions of women’s coping with TFA and assessed to what extent these perceptions are congruent with women’s accounts.

**Methods:**

Fifteen semi-structured interviews were carried out with health professionals in three hospitals in England. Data were analysed using thematic analysis and compared with women’s accounts of their own coping processes to identify similarities and differences.

**Results:**

Health professionals’ perceptions of women’s coping processes were congruent with women’s accounts in identifying the roles of support, acceptance, problem-solving, avoidance, another pregnancy and meaning attribution as key coping strategies. Health professionals regarded coping with TFA as a unique grieving process and were cognisant of women’s idiosyncrasies in coping. They also considered their role as information providers as essential in helping women cope with TFA. The findings also indicate that health professionals lacked insight into women’s long-term coping processes and the potential for positive growth following TFA, which is consistent with a lack of aftercare following TFA reported by women.

**Conclusions:**

Health professionals’ perceptions of women’s coping with TFA closely matched women’s accounts, suggesting a high level of understanding. However, the lack of insight into women’s long-term coping processes has important clinical implications, as research suggests that coping with TFA is a long-term process and that the provision of aftercare is beneficial to women. Together, these findings call for further research into the most appropriate ways to support women post-TFA, with a view to developing a psychological intervention to better support women in the future.

## Background

In England and Wales in 2015, 3213 pregnancies were terminated on the grounds of fetal abnormality representing 2% of all terminations [1]. The number of TFAs has risen (3213 in 2015 compared to 2085 in 2009 [2]), due to increased provision of screening and diagnosis [3], new screening technologies (e.g., non-invasive prenatal testing) that enable earlier fetal abnormality detection, and an increase in childbearing age associated with adverse pregnancy outcomes [4], particularly chromosomal abnormality.

TFA can be a traumatic event with lasting psychological consequences, including posttraumatic stress, complicated grief and depression [5, 6]. Recent studies have highlighted the impact coping strategies have upon the way women adapt to TFA [7, 8]. Strategies considered to be adaptive, such as ‘acceptance’ or ‘positive reframing,’ are generally associated with lower grief levels, whilst strategies considered to be maladaptive, such as ‘denial’ or ‘self-blame,’ generally correlate with higher grief levels [7, 8]. Evidence also indicates that women’s experience of care may relate to their psychological adjustment post-TFA. In particular, compassionate care, i.e., care “given through relationships based on empathy, respect and dignity” ([9], p.13) is greatly valued by women in the context of TFA [10–13].

In England, compassionate care has gained prominence since the publication of the Francis report [14] and the ensuing impetus within the National Health Service to develop a model of care based on compassion and respect. It is also in keeping with the Royal College of Obstetricians and Gynaecologists’ recommendations [15] that women undergoing TFA should receive non-judgemental and supportive care. The ability to provide compassionate care presupposes that health professionals have an understanding of the experience of TFA, the complexity of the decision-making process and the short- and long-term coping processes involved in dealing with it. There is, however, limited evidence on this topic. Existing research underlines challenges experienced by health professionals in caring for women in difficult reproductive situations, including dealing with patients’ emotions [16] or breaking bad news [17, 18]. In the context of TFA, health professionals may also struggle with their own moral dilemmas regarding the decision to terminate on the grounds of fetal abnormality [19], particularly late terminations. These challenges may impede health professionals’ understanding of women’s experience and their ability to provide compassionate care.

Although research suggests that health professionals have some understanding of the emotional difficulties women experience in the context of perinatal loss [16, 20–23], this study is the first to specifically investigate health professionals’ understanding of women’s experience of coping with TFA. The objectives of the study were, therefore, to examine health professionals’ perceptions of women’s coping with TFA and compare them to women’s accounts to assess their congruence.

## Methods

As one of the aims of the study was to compare health professionals’ perceptions of women’s coping with TFA with women’s accounts, both studies are presented in this section in order of completion. The study of women’s accounts is presented first but, as it has been fully reported elsewhere [24], only key methodological information is reported. The study of health professionals’ perceptions is presented second.

### Study of women’s accounts of coping with TFA

Twenty seven women completed online asynchronous semi-structured interviews about their experience of TFA, with an emphasis on the coping strategies used at the time of the procedure and afterwards. Participants were recruited through a support organisation that provides support to parents who face/undergo TFA. The study received ethical approval from the University of West London Ethics Committee in 2011. Women were provided with an information sheet and consent was obtained from all participants. Data were collected by the first author between April 2011 and February 2012. All participants were aged over 18 years old and had experienced TFA. Data were analysed using interpretative phenomenological analysis (IPA). IPA has been widely used in research on reproductive health and perinatal loss [25, 26] and aims to uncover people’s individual experiences and the meanings they attribute to the experiences [27]. The findings indicated that women use several coping strategies to deal with TFA at the time of the procedure and afterwards: ‘seeking/providing support,’ ‘acceptance,’ ‘avoidance,’ ‘problem solving,’ ‘another pregnancy,’ ‘turning to the future’ and ‘meaning attribution.’

### Study of health professionals’ perceptions of women’s coping with TFA

The study of health professionals’ perceptions of women’s coping with TFA, which is the focus of this paper, was conducted as part of a wider research project on the practice of prenatal diagnosis. This project involved observations of fetal medicine consultations and face-to-face interviews with health professionals about their practice. It is in the context of these interviews that health professionals’ perceptions of women’s coping with TFA were explored.

#### Sample

A purposive sampling was used to gain insights from a range of professionals including consultants (or subspecialists) in maternal-fetal medicine, midwives, and sonographers with differing levels of experience. Participants were recruited from three hospitals in England. Sites 1 and 3 were large fetal medicine units in referral centres and teaching hospitals. Site 2 was a local maternity unit providing more limited fetal medicine services such as amniocentesis and chorionic villus sampling. Any health professionals involved in the pregnancy management of women in their unit were eligible to take part. Given that the sites varied in terms of size and services provided, a diversity of professional experience is represented. Participants were recruited by the first author over a 3-week period either face-to-face or via e-mail through a departmental list. Interim analyses of the data indicated that data saturation was reached at 15 interviews; therefore, no further interviews were sought after this point. Of all professionals approached, only one declined to take part citing time constraints.

#### Data collection

Face-to-face semi-structured interviews were conducted at the hospitals between May and July 2013 by the first author. Interviews were recorded digitally. The topic guide focused on health professionals’ perceptions of women’s coping with a diagnosis of fetal abnormality. Questions included: “Could you describe the way you perceive women cope with a diagnosis of fetal abnormality?” “Some women decide to terminate their pregnancy following a diagnosis of fetal abnormality, how do you think they cope with the termination? And afterwards?” Participants were also asked to provide a brief summary of their career path to fetal medicine, and whether they had personal experience of fetal abnormality.

### Data analysis

Data were analysed using thematic analysis (TA). TA has been widely used in reproductive health research [28, 29] and is well-known for its adaptability to different contexts, epistemologies and research questions [30]. TA has also been shown to be compatible with both an inductive (bottom-up) analytical approach, which enables data exploration, and a deductive (top-down) approach, which enables data comparison [30–33]. Given that the study aims were to explore health professionals’ perception of women’s coping with TFA and compare these to women’s accounts, TA was an appropriate method of data analysis for this study. The analytical process first consisted of identifying themes in the health professionals’ data using an inductive (bottom-up) approach. This enabled the research team to explore and preserve the originality and richness of individuals’ accounts. Data were also analysed using a deductive (top-down) approach, using the coding framework derived from the women’s data, to explore health professionals’ perceptions of women’s coping with TFA and to assess how congruent these perceptions were with women’s accounts.

Data were transcribed verbatim, line-numbered and analysed by the first author. The analytical process followed Braun and Clarke’s guidelines [30] involving: data familiarisation, generation of initial codes, identification of themes, revision and refinement of themes, definition and naming of themes, and report writing. To ensure that the full range of participants’ accounts was represented, care was taken not to force the data into existing codes. Disconfirming cases, whereby evidence that contradicts an established or emergent code is sought and coded [34], were also included in the analysis. To enhance rigour and validity, sections of text were independently coded by the fourth author. The level of agreement between the two researchers was high and no changes were made to the coding framework. The third author provided expert validation of the analysis.

#### Ethics

Ethical approval for the research project into health professionals’ practice of prenatal diagnosis, of which the present study is a part, was received from the University of West London Ethics Committee (July 2012) and the Stanmore NHS Research Ethics Committee (February 2013). All participants were provided with an information sheet and made aware of their rights regarding confidentiality and withdrawal from the study. Consent for taking part in the study and the use of quotations was obtained from all participants; all were sent a summary of the results. The researcher who conducted the fieldwork had no professional relationship with the participants. As such, there was no conflict of interest.

## Results

### Participants’ profile

Fifteen health professionals participated in the study, ten from Site 1, four from Site 2 and one from Site 3. The sample comprised six consultants (subspecialists) in maternal-fetal medicine or genetics, one registrar (resident) in fetal medicine, four midwives (including a senior screening coordinator), two sonographers, one specialist nurse and one healthcare assistant. Twelve participants were female and three were male. Their age ranged between 24 and 55 years old.

The Results section is organised in two parts. Part 1 focuses on the themes identified through the inductive analysis and depicts health professionals’ reflections about women’s coping with TFA and their role in supporting women. Part 2 focuses specifically on health professionals’ perceptions of the coping strategies used by women. These themes were identified through the deductive process of analysis using the coding framework generated from the women’s data. Illustrative quotations are attributed to participants as follows: consultants (C), registrar (Reg), midwives (Mid), sonographers (Son), specialist nurse (SNur) and healthcare assistant (HCA).

### Part 1 - health professionals’ reflections about women’s coping with TFA and their role in supporting women

The inductive analysis identified four themes: ‘the nature of coping with TFA,’ ‘the idiosyncrasies of women’s coping,’ ‘helping women cope’ and ‘the limitations to health professionals’ understanding of women’s long-term coping.’ Each theme encompassed several subthemes.

### The nature of coping with TFA

#### TFA as a unique grieving process

Health professionals considered TFA as a unique grieving process that can be difficult and lengthy: “*I do see them [the women] post-termination and they may be in tears the whole consultation, they’re struggling, they haven’t coped with the problem*” (C3) and may generate a range of emotions such as “denial,” “anger,” and eventually, “acceptance” (C1). Some professionals considered the grieving process to start from the time of diagnosis: “*You do lose something of that concept of a healthy baby, even if ultimately your decision is to continue*” (C1). Difficulty with grieving could extend well beyond the termination and be manifest during subsequent pregnancies: “*They don’t cope because it’s a grieving process that goes on for a long time, and even when they come back for the next pregnancy, they bring it up*” (Mid1).

Participants regarded the loss of the baby as central to the grieving process, however they also commented on the loss of potential, which some considered to be harder to deal with:

*With the baby you’re grieving for the loss of potential, and I think it’s harder to grieve for that loss of potential than it is for somebody who you can say, well they had a good innings* (C2).

#### The burden of choice

Participants considered coping with TFA to be complicated by the fact that women made the decision to end the pregnancy. References to women experiencing guilt were common: “*I think everybody probably feels guilty because you never know whether it was the right decision*” (SNur). This was, however, contrasted by one professional who considered the decision to terminate as secondary to the grieving process, likening the experience to coping with a stillbirth:

*I mean it’s like a stillbirth isn’t it?…it’s actually very difficult to distinguish women who have an unexpected stillborn but normal baby from women who have had a baby that had abnormality they have had to kill* (C1)*.*

Health professionals believed the guilt experienced by some women to relate to the decision to terminate but also to causing the abnormality in the first place: “*They always want to think had I done this or had I done that, or did I do this, should I have come to the hospital earlier, should I had eaten this*” (Mid1). In addition, some professionals mentioned women’s fear of being judged: “*Parents feel that they might be judged by other people around them, especially if they’re saying that they’ve had a termination*” (Mid1). In some cases, they reported women justifying their decision to them: “*They will try and justify it [the decision] to make it seem better, I guess, for them*” (Mid6).

### The idiosyncrasies of women’s coping

All participants acknowledged that “*people are very different*” (Son2) and that coping is an individual process.

#### Personal characteristics

Women’s personality and beliefs, coping styles and capacity for self-regulation were seen as influential in determining the way they cope with TFA. How *“sentimental” or “emotional”* (C1) a woman is was thought to relate to her willingness or ability to seek support, which in turn could influence the way she coped*:* “*if you’re someone that is very much not emotional, and keeping … keep it quite clos*e” (Mid2). Religious beliefs were also seen as important: “*There are some people who just think that’s God’s will and they accept it and they just cope with it*” (Son2). Differences in coping styles could also influence women’s coping:

*Some people are very actively involved and sort of want to know everything and, you know, even want to see the baby after birth and take pictures etcetera and other people just sort of want to have nothing to do with it* (C1).

Several professionals also noted differences in women’s capacity for self-regulation and their support needs:

*I don’t think everyone necessarily wants huge amounts of support shoved at them because I think that in a way it can become counter-productive as well. You know if your coping mechanisms are working…then actually having huge amounts of leaflets and follow-up phone calls and counselling visits and whatever aren’t necessarily helpful* (Reg).

#### Termination-related variables

Termination-related variables were also thought to influence women’s coping. Some professionals considered that an advanced gestational age could complicate the coping process. Referring to a woman who was 36 weeks pregnant when terminating her pregnancy, the specialist nurse commented: “*I think her being so late on in her pregnancy is going to have a massive effect on how she copes now*” (SNur). However, other professionals considered an early loss to be equally, if not more, difficult to cope with:

*I think it’s equally hard to lose a baby at 12 weeks as it is at 9 months…People who have an early loss, often have a harder time because everyone else presumed it’s easier to lose a baby at 12 weeks than it is at 9 months* (C2).

The baby’s prognosis could also influence the way women cope, the assumption being that it may be easier to cope with TFA when the prognosis is incompatible with life: “*I think certain conditions, it’s easier for them to cope with… we have a lady, the baby had acrania, so obviously she was devastated but she understood that the baby was not compatible with life*” (Mid3).

#### Women’s environment

Women’s environment such as family situation, culture and beliefs were also thought to impact upon the way they cope. Having a “*close-knit support system*” (C6) was seen as important alongside having children to look after: “*having other children helps them to cope…they’ve got a little child, smiley happy child that keeps them busy, distracted*” (Mid 10). Cultural differences in attitudes towards adversity were also reported as a factor. One professional suggested that women in the UK may feel pressured into hiding their emotions: “*Pressure on them to stiff upper lip, be British and not cry*” (Son1).

### Helping women cope

#### Providing information that enables women to reach the right decision

Participants regarded their primary role as providing information that enables women to reach the right decision regarding their pregnancy. They believed that women’s chances of adjusting well to TFA were increased if they felt that they had made the decision most appropriate to their circumstances: *“[what helps women cope is] knowing that it’s the right thing because either the baby wouldn’t have survived or would have been distressed so they just keep on reminding themselves that this is the right thing to do”* (C4). Consequently, providing women with clear, comprehensive and balanced information was seen as paramount by professionals.

Sonographers were usually the first to detect an abnormality, but recognised that the diagnosis and counselling about the pregnancy management remained within the consultants’ remit. However, being at the forefront of the process of identifying fetal abnormalities, sonographers were acutely aware of their responsibility in delivering the news in the most appropriate way: “*I think how they cope is depending on how your behaviour is as a sonographer....I think if you’re scared as a sonographer in how you’re going to deliver this news, you won’t deliver it well*” (Son1).

Consultants considered the provision of balanced information as their key responsibility: “*My responsibility is to make sure they’re making the right decision for them*” (C4). “*[I explained the problem] but I also explain to them what the termination would involve, because people don’t have any idea, they don’t know that they’re going to go through a feticide*” (C3). One consultant referred to his role as a guide through a shared decision-making process: “*It’s not me counselling the parents. It’s us having a conversation almost about what we’ve found and making a decision together, and agreeing an action plan*” (C1). However, this was an isolated comment.

Midwives also provided information to women on diagnostic tests, termination procedure or fetal conditions. They too emphasised the importance of providing balanced information: “*The way you counsel a woman, you tell her the whole picture, you tell her everything about that situation, whether you feel biased about it or not*” (Mid1). Information provision also involved directing women to organisations or support groups that could complement the information received at the hospital.

#### Providing emotional support

Health professionals also saw their role as supporting women emotionally, but this was usually considered secondary to the information provision. This role was mainly imparted to the midwives: “*[the midwives] potentially will see them [the women] and they’ll speak to them on phone, and they are very good and I think quite often they will phone and see how they are doing*” (Reg). Midwives were aware of the importance of supporting women through this difficult time and saw empathy as essential: “*I can give them the best that I can and be as supportive as I can. I can’t change it for them. I can only try and make it bearable*” (Mid4);

*I couldn’t just sit here to you… and you’re crying your eyes out, and I’m sitting here watching you so I would have to get up from my seat and come round to you…they need the time, they need the empathy* (Mid1).

Health professionals also viewed their role as alleviating women’s possible feelings of guilt: “*I always try to say to them, coming back to the consultation, that’s not their fault…it’s one of those things*” (C3).

#### The aftercare

Aftercare was regarded as an important factor in women’s coping processes, but participants recognised that it is “patchy” (Reg). Professionals commented on how isolated women may feel after the termination: “*I often feel when they have a termination and they just go home and they’re left to deal with it*” (SNur). This could contrast the intense level of care women receive up to the termination:

*There is a sort of cliff-edge effect…you find a baby with an abnormality and you see them every week in the fetal medicine unit, and you get counselled by 6000 people and then you have a termination and then go home and you don’t see anyone anymore* (C1).

Health professionals were unclear on what aftercare should consist of, how it should be delivered and by whom: “*They might not want to be just randomly called by someone they’ve met once or twice*” (SNur); “*I don’t necessarily think we are the right people to provide support afterwards, because quite often people do not want to come back here*” (Reg). They were also aware that some women may find it difficult to resume contact with them after the termination and/or during subsequent pregnancies: “*I always ask the woman, do you want to see me again or do you want to see somebody else*” (Mid1).

### Limitations to health professionals’ understanding of women’s long-term coping

All participants acknowledged limitations to their understanding of women’s long-term coping with TFA. As participants in this study worked in secondary or tertiary care settings, their interactions with women did not usually go beyond the termination or the follow-up appointment: “*They [the women] are going to go off and you don’t see them again*” (Reg). “*We’re focused on the immediate, but actually it’s the immediate to long-term changes and I, you and I, have no access to that in general*” (C2). Furthermore, participants intervened at different points in the TFA process. Sonographers were usually the first to detect an abnormality but usually did not see women beyond this point. Consultants were involved in the diagnosis and the procedure (if a feticide was required), but often did not see women after the termination:

*How they cope, I don’t know. Do you? I don’t go home with them, I don’t know who their friends are, I don’t know who their family are, I don’t know whether they take drugs, I don’t know whether they have hobbies, I have no idea how they cope* (C2).

Seeing women at their follow-up appointment did not necessarily provide consultants with insights into the way women coped: “*Apart from seeing these women for a sort of debrief, I don’t know what’s going on in the background. I don’t know how much care they are getting from the GPs or from midwives here in the hospital*” (C5). The only exception was the genetic counsellor who could, in some instances, have a longer-term involvement with women.

Midwives generally had a broader understanding of women’s long-term coping processes from seeing women from their first appointment to after the termination. Midwives coordinating women’s care after discharge, generally had a longer-term involvement with the women:

*There’s one lady that had a really difficult one, late diagnosis, at 37 weeks, and I still talk to her at least two or three times a week sometimes, until she has that postmortem, she just can’t stop calling, and I understand that* (Mid1).

### Part 2 - health professionals’ perceptions of women’s coping strategies

This section focuses specifically on health professionals’ perceptions of women’s coping strategies when dealing with TFA. For this part of the analysis, the coding framework generated from the women’s accounts [24] was used to code the health professionals’ data. The analysis identified six themes present in both the women and the professionals’ accounts: ‘support,’ ‘acceptance,’ ‘problem solving,’ ‘avoidance,’ ‘another pregnancy’ and ‘meaning attribution.’

### Support

#### Individual-based support

Professionals identified women’s partners as an essential source of support, particularly on the day of the termination: “*They’re often more physically approximate [close] to their partner than [they] ever been before....when they come for their termination, they’re often sitting together and holding hands*” (C2). Several participants also commented on women supporting their partner through the process: “*I think at the time that it happens both seem to be in the same place of grieving and just getting through this, so they support each other a lot*” (C6). Support from friends and relatives could also help women: “*I think it’s really supportive to have your own family and friends that can be there to just pick you up*” (Mid4). Religious support was also cited as a source of support; this could involve using spiritual items during the procedure such as candles, saying prayers, or having the baby blessed immediately after the birth:

*I took a lady, it was very late [in pregnancy], but she came along with a friend and they brought a candle, and they sat there and they were saying some prayers, and then I took them afterwards to the chapel and they said some prayers* (HCA).

#### Professional-based support

Health professionals believed that the support they provide to women also influences women’s coping: “*The staff obviously, the midwives that are looking after you, they tend to be supportive and not judgemental, just being there to get you through the best that we can*” (Mid4). Professional support could also take the form of counselling: “*They sometimes have looked for help. They have seen a counsellor*” (C3). Health professionals acknowledged, however, that counselling may not be appropriate for all women: “*I know some people will take up counselling, some people are not ready for it when we first see them*” (SNur). Professional support also included the use of support organisations. Most participants spontaneously mentioned several organisations they considered to be an effective source of support for women: “*I hope maybe by going on a website or a support group or something, that [seeking support] is much easier to do. You can do it in the evening at home, they may do that*” (C6).

### Acceptance

#### Accepting the situation

Some professionals, particularly midwives, considered acceptance of the situation as a prerequisite of coping: “*If you acknowledge it, you can grieve. If you don’t acknowledge it, you just can’t*” (Mid2). Having the opportunity to talk about their situation was regarded as beneficial to women. However, professionals believed that they, themselves, were not necessarily the most appropriate source of support:

*Sometimes people want to talk to other people who have been through the same… some people don’t want to have that connection with the hospital, they want somebody completely separate* (HCA).

#### Acknowledging the baby

Acknowledging the baby was regarded as important to women’s coping. This could involve naming the baby: “*They gradually get over it. Some of them, they’ve named that baby, especially if they’ve terminated*” (Mid1). Taking pictures and using a memory box were also seen as helpful:

“*They [at the hospital] do it very well, the pictures are nice…They give that to a patient so they’ve got a little bit of a picture and the blanket that they [the baby] were wrapped in, for some people that’s really nice…They want to go home and they want to have something with them that reminds them of what was*” (HCA).

Attending a “*remembrance service*” (Mid4) or having a burial were also considered to be helpful in providing women with a sense of closure: “*I think if they went for cremation, for example, many people find that very helpful. Again it helps a little bit with grieving and closure*” (C6). Celebrations of milestones such as birthdays were also reported as beneficial.

### Problem solving

Adopting a practical ‘problem-solving’ approach to the termination was another of women’s coping strategies reported by health professionals. This involved gathering information: “S*ome people will cope by trying to fix or solve the problem, and will go and get a second opinion or do lots of research, or come up with lots of information*” (Reg). Some professionals also regarded this approach as one way for women to regain some control over the situation: “*I think many people will seek more information to try and get a bit more control over, understanding it better because this thing is thrown at them*” (C6). Similarly, several professionals reported women wishing to be involved in the termination procedure, expressing a desire to look at the screen during the feticide and requesting photographs of the baby:

*In general we turn off the screens [during feticide] and I know, they don’t want to see what we’re doing, but you know people are different. Some people have asked to see…and some people will ask for images and pictures and stuff, even at the time of termination* (C2).

Problem solving could also involve focusing on the medical task and regarding the procedure as a medical intervention to go through rather than reflecting on its meaning: “*Think about it as a procedure, that this is what I need to do, you’re going to give me instruction*” (Mid2).

### Avoidance

Several avoidant strategies used by women at the time of the procedure were also reported by health professionals, such as avoiding information: “*We have a few people who say on purpose they didn’t read up at all. They don’t want to know more*” (C6). Avoidance was also manifest in women’s attempts to dissociate from the procedure: “*[some people] close their eyes and say ‘you know, just let me know when it’s all done and then we can get out of here*’” (C1). Avoidant strategies were also reported post-termination. Health professionals described women’s attempts to keep busy, with some women returning to work quickly after the termination: “*I’ve seen a woman today who wants to go back to work as soon as possible…because she feels she will cope better if she’s busy*” (C5). Actively avoiding thinking about the termination was also mentioned: “*I think there are some people who actually want to put that behind them and don’t want to be reminded of it*” (Son2).

### Another pregnancy

#### The focus on another pregnancy

Another pregnancy was seen by health professionals as a way of coping for women: “*Most people want to get on with the next pregnancy straight away actually*” (C4). Planning another pregnancy was regarded as an indication of women’s readiness to move on: “*I say to them [the women] if you feel psychologically ready then there is no advantage of waiting. I think that [for] most people it’s better for them to get on and try again*” (C4). Similarly, a positive pregnancy outcome could be regarded as an indication that women had adjusted well to the termination: “*I think most women seem to recover quite well when they’ve had a happy pregnancy outcome*” (C5). One professional, however, raised potential issues with seeking a new pregnancy too quickly after the termination in what he described as a “*replacement strategy*” (C1).

#### Challenges in caring for women in subsequent pregnancies

Participants acknowledged that a subsequent pregnancy is a time of heightened anxiety for women, during which they relive their difficult experience: “*When they’re booking that scan, they will get all emotional. And like a choking thing, it’s all coming back again*” (Mid1). Sensitive care in subsequent pregnancy was, therefore, considered as paramount: “*I think giving them supportive care in subsequent pregnancies, I think is very important*” (C5). However, participants commented on the challenges in managing women’s levels of anxiety during subsequent pregnancies:

*They’re left in this limbo, although we give them the risk of re-occurrence being quite low, I think they focus on what the, if you reverse, you know 96% likely you’ll be absolutely fine; they still can’t focus on that. They’ve been in that 4%* (SNur).

### Meaning attribution

Meaning attribution as a potential coping strategy was seldom mentioned by participants. However, one professional reported some women undergoing a medical termination (which necessitates giving birth to the baby), deriving meaning from the birth experience: “*A few women who I’ve seen for counselling afterwards have said that whilst the experience was unpleasant, horrible or awful, they’ve felt that they needed to go through it to sort of as part of their grieving or something*” (C5).

## Discussion

This study sought to examine health professionals’ perceptions of women’s coping with TFA and compare these to women’s accounts of their own coping processes. The findings indicate that health professionals’ perceptions of women’s coping covered six areas that were also present in women’s accounts: ‘support,’ ‘acceptance,’ ‘problem solving,’ ‘avoidance,’ ‘another pregnancy’ and ‘meaning attribution.’ In addition, the inductive analysis identified four themes relating to professionals’ reflections about coping with TFA and their role in supporting women: ‘the nature of coping with TFA,’ ‘the idiosyncrasies of women’s coping,’ ‘helping women cope’ and ‘the limitations to health professionals’ understanding of women’s long-term coping.’

### Congruence between health professionals’ and women’s accounts

A comparison between the health professionals’ and women’s data identified more commonalities than differences. Both groups regarded coping with TFA as a unique grieving process, which can be complex and lengthy. Health professionals also alluded to limitations to their understanding of women’s longer-term coping with TFA, and to inadequacies in the aftercare offered to women. This was consistent with women’s accounts of limitations in the aftercare [24]. The findings also indicate that health professionals’ perceptions of women’s coping were mostly congruent with women’s accounts. Both groups of participants considered ‘support,’ ‘acceptance,’ ‘problem solving’ and ‘another pregnancy’ as important strategies for women in coping with TFA.

The comparative analysis also revealed some differences. Making the most appropriate decision was identified by the health professionals as a key factor in women’s coping process. Health professionals were forthcoming in describing their role in helping women make that decision and viewed their role primarily as information provider. In comparison, women seldom mentioned their decision and when commenting on health professionals’ involvement, it was mainly as providers of emotional support. Another difference relates to the importance of ‘meaning attribution,’ a theme far more prominent in women’s accounts than in the professionals’ data. Similarly, the theme ‘looking to the future’ identified in the women’s data, which encompasses the positives of the decision to terminate, putting the experience to good use and deriving personal growth, was not represented in health professionals’ accounts.

Despite these differences, overall the findings suggest a high level of congruence between women’s and health professionals’ perspectives on women’s coping with TFA. This implies that health professionals, in this study, had a valid understanding of what coping with TFA involves for women. This may have significant clinical implications as understanding patients’ experience may assist health professionals in delivering person-centred care. In turn, this may increase satisfaction with care and promote better health outcomes for women. This is consistent with evidence suggesting that women’s experience of healthcare when undergoing TFA may influence the way they adjust to it [10–13]. It is also pertinent within the context of clinical concordance and shared decision making that has been shown to improve patients’ health outcomes [35, 36].

Shared decision making may be particularly important in the context of TFA as the decision to continue or end the pregnancy is irreversible and has important psychosocial consequences. In the context of perinatal or neonatal loss, parents rely to a greater extent upon health professionals than their close family and friends to make decisions [37], possibly because of health professionals’ technical expertise, but also in an attempt to share ‘the burden of choice.’ Evidence suggests that women uncomfortable with their decision experience higher levels of psychological distress than those who report being comfortable with it [7]. Thus, it is paramount that women reach a decision that is appropriate to their circumstances. The health professionals in this study were acutely aware of this imperative and accordingly viewed their role as information provider, a responsibility consistent with clinical guidelines in the UK [38].

### The need for aftercare

The findings also reveal a lack of insights into the way women cope long-term with TFA, which reflects health professionals’ limited clinical involvement with the women. It is consistent with a perceived lack of aftercare reported in women’s accounts [24] and in the TFA and wider perinatal loss literature [10, 11, 20–23, 39, 40]. The lack of aftercare may have important implications for women’s long-term adjustment post-TFA. However, the nature of the type of aftercare needed remains to be ascertained. A recent study into the support desired by women who undergo TFA indicates that, at the time of the termination, most women have not anticipated what their support needs may be [41]. It is also unrealistic to expect health professionals responsible for women’s care at the time of termination to provide the entirety of the aftercare. Furthermore, as indicated in this study, women may prefer to be cared for post-termination by professionals not associated with the termination, and may favour support they can access as and when needed. Research has identified important milestones in women’s grieving following TFA, such as the baby’s due date or the first anniversary [24]. Therefore, women’s need for support increases at specific points.

Whether psychosocial interventions would be effective in supporting women who struggle to adjust post-TFA also warrants investigation. Cognitive Behavioural Therapy (CBT) based interventions following perinatal loss have been shown to reduce depression and anxiety [42, 43]. In the context of TFA, evidence of the effectiveness of psychological interventions post-termination is inconclusive and most of it is dated [44, 45]. Nevertheless, a more recent study by Kersting et al. [46] into the effectiveness of a CBT web-based intervention following perinatal loss, which included a small sample of women who had undergone TFA, shows promising results. Those receiving the intervention exhibited fewer symptoms of posttraumatic stress, grief, depression, and anxiety, with the benefits still apparent at 3- and 12-months post-treatment. Despite these encouraging results, further research focusing exclusively on women who have undergone TFA would be needed to ascertain whether such intervention would be effective with this particular group of women.

### TFA as unique form of bereavement

The findings indicate that both health professionals and women consider TFA as a unique form of bereavement, in keeping with existing literature [40], and to the relevance of bereavement theories. First, there was evidence that maintaining bonds with the baby were soothing for women and acknowledged as such by professionals. This is consistent with the Continuing Bonds theory [47] that posits that the purpose of grief is not to sever bonds with the deceased but to maintain and integrate them with others. Second, the importance of ‘meaning attribution’ to women points to the relevance of the bereavement model of ‘finding meaning’ [48] that posits that following bereavement, individuals need to engage in the task of reconstructing meaning in a way that is congruent with their situation and experience. Research has shown that both theories are highly relevant to the context of bereavement [49, 50] and the present study supports this.

### The potential for positive growth

The findings also indicate that health professionals do not anticipate women’s potential for positive growth following TFA. Positive growth refers to positive changes that occur following a traumatic event and may involve transformations on personal, philosophical and interpersonal dimensions [51, 52]. Coping strategies reported by women such as ‘looking to the future,’ which included deriving personal growth, were not mentioned by health professionals, while ‘meaning attribution’ was only mentioned by one. This is despite research suggesting that positive growth is an important element in women’s coping in the context of TFA and, more widely, perinatal loss [40, 53]. This is also consistent with the posttraumatic growth literature suggesting that individuals may experience positive growth following a traumatic event [51–53]. Health professionals need to be cognisant of this phenomenon in the context of TFA and consider ways to promote it.

Recent studies on TFA have shown that ‘acceptance’ and ‘positive reframing’ relate to lower grief levels [7] and that positive reframing predicts the experience of positive growth [54]. Interventions that promote acceptance and reframing may, therefore, be beneficial to women in encouraging the creation of new narratives.

### Limitations of the study

The study has several limitations. The fieldwork was mostly conducted in two hospitals in England, therefore, the findings may not be transferrable to other units or healthcare systems. The hospital in which most of the interviews were conducted (Site 1) is a large fetal medicine centre. Therefore, the staff’s level of expertise and the profile of the cases they manage may differ from other fetal medicine centres. Health professionals working in smaller units and managing cases not requiring referral to tertiary units, may be involved in women’s care for longer periods of time and consequently, have a more in-depth understanding of women’s long-term coping with TFA.

The sample was self-selected, which raises the potential for bias. Furthermore, these interviews were conducted as part of a larger research project that involved observations of clinical consultations. Most of the professionals who participated in the interviews were also observed by the first author during clinical consultations. Therefore, a degree of social desirability bias cannot be excluded.

## Conclusion

This study is the first to assess health professionals’ perceptions of women’s coping with TFA. It demonstrates a high level of congruence between health professionals’ and women’s accounts of women’s coping processes. However, the study also reveals a lack of understanding of women’s long-term experience of coping. This is consistent with women’s reports of a lack of aftercare following TFA and, more generally, perinatal loss. This has important implications for the optimisation of women’s health outcomes following TFA. Further research into the type of aftercare needed following TFA and the modalities of its delivery is necessary, with the aim being to develop an intervention for women in need of support post-TFA The study also indicates the potential for positive growth following TFA, emphasising the importance of adopting a ‘woman-centred’ rather than ‘one size fits all’ approach to care provision.

## Abbreviations

CBT: Cognitive behavioural therapy
IPA: Interpretative phenomenological analysis
TA: Thematic analysis
TFA: Termination of pregnancy for fetal abnormality

## References

[1] Department of Health*.* Abortion statistics, England and Wales in 2015. 2016. https://www.gov.uk/government/uploads/system/uploads/attachment_data/file/570040/Updated_Abortion_Statistics_2015.pdf. Accessed 5 Jan 2017.

[2] Department of Health. Abortion statistics for England and Wales in 2009 (2010–2015). https://www.gov.uk/government/uploads/system/uploads/attachment_data/file/216085/dh_116336.pdf (2010–2015). Accessed 5 Jan 2017.

[3] Nicolaides KH (2011). A model for a new pyramid of prenatal care based on the 11 to 13 weeks’ assessment. Prenat Diagn.

[4] Royal College of Obstetricians and Gynaecologists. RCOG statement on later maternal age. London: RCOG; 2009. https://www.rcog.org.uk/en/news/rcog-statement-on-later-maternal-age/. Accessed 8 Mar 2015.

[5] Kersting A, Kroker K, Steinhard J, Hoernig-Franz I, Wesselmann U, Luedorff K (2009). Psychological impact on women after second and third trimester termination of pregnancy due to fetal anomalies versus women after preterm birth—a 14-month follow up study. Arch Womens Ment Health.

[6] Korenromp MJ, Page-Christiaens GC, van den Bout J, Mulder EJ, Visser GH (2009). Adjustment to termination of pregnancy for fetal anomaly: a longitudinal study in women at 4, 8, and 16 months. Am J Obstet Gynecol.

[7] Lafarge C, Mitchell K, Fox P (2013). Perinatal grief following a termination of pregnancy for foetal abnormality: the impact of coping strategies. Prenat Diagn.

[8] Nazaré B, Fonseca A, Canavarro MC (2013). Adaptive and maladaptive grief responses following TOPFA: actor and partner effects of coping strategies. J Reprod Infant Psychol.

[9] Cummings J, Bennett V. Compassion in practice. Nursing, midwifery and care staff. Our vision and strategy. NHS Commissioning Board. 2012. https://www.england.nhs.uk/wp-content/uploads/2012/12/compassion-in-practice.pdf. Accessed 5 Apr 2014

[10] Asplin N, Wessel H, Marions L, Georgsson Öhman S (2014). Pregnancy termination due to fetal anomaly: women’s reactions, satisfaction and experiences of care. Midwifery.

[11] Fisher J, Lafarge C (2015). Women’s experience of care when undergoing termination of pregnancy for fetal anomaly in England. J Reprod Infant Psycho.

[12] McCoyd JL (2009). What do women want? Experiences and reflections of women after prenatal diagnosis and termination for anomaly. Health Care Women Int.

[13] Statham H (2002). Prenatal diagnosis of fetal abnormality: the decision to terminate the pregnancy and the psychological consequences. Fetal Matern Med Rev.

[14] The Mid Staffordshire NHS Foundation Trust Public Inquiry (2013). Report of the Mid Staffordshire NHS Foundation Trust Public Inquiry: executive summary.

[15] Royal College of Obstetricians and Gynaecologists. Termination of pregnancy for fetal abnormality in England, Scotland and Wales. Report of a Working Party. 2010. https://www.rcog.org.uk/globalassets/documents/guidelines/terminationpregnancyreport18may2010.pdf. Accessed 27 Nov 2014.

[16] Menezes MA, Hodgson JM, Sahhar M, Metcalfe SA (2013). “Taking its toll”: the challenges of working in fetal medicine. Birth.

[17] Guerra FAR, Mirlesse V, Baião AER (2011). Breaking bad news during prenatal care: a challenge to be tackled. Cien Saude Colet.

[18] Lalor JG, Devane D, Begley CM (2007). Unexpected diagnosis of fetal abnormality: women’s encounters with caregivers. Birth.

[19] Fay V, Thomas S, Slade P (2016). Maternal-fetal medicine specialists’ experiences of conducting feticide as part of termination of pregnancy: a qualitative study. Prenat Diagn.

[20] Geller PA, Psaros C, Kornfield SL (2010). Satisfaction with pregnancy loss aftercare: are women getting what they want?. Arch Womens Ment Health.

[21] Gold K (2007). Navigating care after a baby dies: a systematic review of parent experiences with health providers. J Perinatol.

[22] Kelley MC, Trinidad SB (2012). Silent loss and the clinical encounter: parents’ and physicians’ experiences of stillbirth–a qualitative analysis. BMC Pregnancy Childbirth.

[23] Rowlands IJ, Lee C (2010). ‘The silence was deafening’: social and health service support after miscarriage. J Reprod Infant Psycho.

[24] Lafarge C, Mitchell K, Fox P (2013). Women’s coping with pregnancy termination for fetal abnormality: an interpretative phenomenological analysis of women’s experiences. Qual Health Res.

[25] Shaw RL (2011). Women’s experiential journey toward voluntary childlessness: an interpretative phenomenological analysis. J Community Appl Soc Psychol.

[26] Ryninks K, Roberts-Collins C, McKenzie-McHarg K, Horsch A (2014). Mothers’ experience of their contact with their stillborn infant: an interpretative phenomenological analysis. BMC Pregnancy Childbirth.

[27] Smith JA, Flowers P, Larkin M (2009). Interpretative phenomenological analysis: theory, method and research.

[28] Hadjigeorgiou E, Coxon K (2013). In Cyprus, ‘midwifery is dying…’. A qualitative exploration of midwives’ perceptions of their role as advocates for normal childbirth. Midwifery.

[29] Malik SH, Coulson NS (2008). Computer-mediated infertility support groups: an exploratory study of online experiences. Patient Educ Couns.

[30] Braun V, Clarke V (2006). Using thematic analysis in psychology. Qual Res Psychol.

[31] Joffe H, Yardley L, Marks DF, Yardley L (2004). Content and thematic analysis. Research methods for clinical and health psychology.

[32] Coulson NS (2005). Receiving social support online: an analysis of a computer-mediated support group for individuals living with irritable bowel syndrome. Cyberpsychol Behav.

[33] Fereday J, Muir-Cochrane E (2006). Demonstrating rigor using thematic analysis: a hybrid approach of inductive and deductive coding and theme development. Int J Qual Methods.

[34] Pope C, Ziebland S, Mays N (2000). Qualitative research in health care: analysing qualitative data. BMJ.

[35] De las Cuevas C, Rivero-Santana A, Perestelo-Perez L, Perez-Ramos J, Serrano-Aguilar P (2012). Attitudes toward concordance in psychiatry: a comparative, cross-sectional study of psychiatric patients and mental health professionals. BMC Psychiatry.

[36] Joosten EA, DeFuentes-Merillas L, de Weert GH, Sensky T, van der Staak CP, de Jong CA (2008). Systematic review of the effects of shared decision-making on patient satisfaction, treatment adherence and health status. Psychother Psychosom.

[37] Williams C, Munson D, Zupancic J, Kirpalani H (2008). Supporting bereaved parents: practical steps in providing compassionate perinatal and neonatal end-of-life care–a north American perspective. Semin Fetal Neonatal Med.

[38] National Institute for Health and Care Excellence. Antenatal care routine care for the healthy pregnant woman. 2008. https://www.nice.org.uk/guidance/cg62. Accessed 4 Apr 2015.

[39] Koponen K, Laaksonen K, Vehkakoski T, Vehmas S (2013). Parental and professional agency in terminations for fetal anomalies: analysis of Finnish women’s accounts. Scan J Disab Res.

[40] Lafarge C, Mitchell K, Fox P (2014). Termination of pregnancy for fetal abnormality: a meta-ethnography of women’s experiences. Reprod Health Matters.

[41] Ramdaney A, Hashmi SS, Monga M, Carter R, Czerwinski J (2015). Support desired by women following termination of pregnancy for a fetal anomaly. J Genet Couns.

[42] Bennett SM, Ehrenreich-May J, Litz BT, Boisseau CL, Barlow DH (2012). Development and preliminary evaluation of a cognitive-behavioral intervention for perinatal grief. Cogn Behav Pract.

[43] Séjourné N, Callahan S, Chabrol H (2010). The utility of a psychological intervention for coping with spontaneous abortion. J Reprod Infant Psycho.

[44] Elder S, Laurence K (1991). The impact of supportive intervention after second trimester termination of pregnancy for fetal abnormality. Prenat Diagn.

[45] Lorenzen J, Holzgreve W (1995). Helping parents to grieve after second trimester termination of pregnancy for fetopathic reasons. Fetal Diagn Ther.

[46] Kersting A, Dolemeyer R, Steinig J, Walter F, Kroker K, Baust K (2013). Brief internet-based intervention reduces posttraumatic stress and prolonged grief in parents after the loss of a child during pregnancy: a randomized controlled trial. Psychother Psychosom.

[47] Klass D, Silverman PR, Nickman SL (1996). Continuing bonds: new understandings of grief.

[48] Neimeyer RA (2001). Meaning reconstruction & the experience of loss.

[49] Keesee NJ, Currier JM, Neimeyer RA (2008). Predictors of grief following the death of one’s child: the contribution of finding meaning. J Clin Psychol.

[50] Neimeyer RA, Baldwin SA, Gillies J (2006). Continuing bonds and reconstructing meaning: mitigating complications in bereavement. Death Stud.

[51] Joseph S (2011). What doesn’t kill us makes us stronger: the new psychology of posttraumatic growth.

[52] Tedeschi RG, Calhoun LG (2004). Posttraumatic growth: conceptual foundations and empirical evidence. Psychol Inq.

[53] Black B, Sandelowski M (2010). Personal growth after severe fetal diagnosis. West J Nurs Res.

[54] Lafarge C, Mitchell K, Fox P (2017). Posttraumatic growth following pregnancy termination for fetal abnormality: the predictive role of coping strategies and perinatal grief. Anxiety Stress Coping.

